# Epigenetic Switch at Atp2a2 and Myh7 Gene Promoters in Pressure Overload-Induced Heart Failure

**DOI:** 10.1371/journal.pone.0106024

**Published:** 2014-09-02

**Authors:** Tiziana Angrisano, Gabriele Giacomo Schiattarella, Simona Keller, Gianluigi Pironti, Ermanno Florio, Fabio Magliulo, Roberta Bottino, Raffaela Pero, Francesca Lembo, Enrico Vittorio Avvedimento, Giovanni Esposito, Bruno Trimarco, Lorenzo Chiariotti, Cinzia Perrino

**Affiliations:** 1 Department of Molecular Medicine and Medical Biotechnology, Federico II University, Naples, Italy; 2 Department of Biology, Federico II University, Naples, Italy; 3 Department of Advanced Biomedical Sciences, Federico II University, Naples, Italy; 4 Department of Medicine, Duke University Medical Center, Durham, North Carolina, United States of America; Cleveland Clinic, United States of America

## Abstract

Re-induction of fetal genes and/or re-expression of postnatal genes represent hallmarks of pathological cardiac remodeling, and are considered important in the progression of the normal heart towards heart failure (HF). Whether epigenetic modifications are involved in these processes is currently under investigation. Here we hypothesized that histone chromatin modifications may underlie changes in the gene expression program during pressure overload-induced HF. We evaluated chromatin marks at the promoter regions of the sarcoplasmic reticulum Ca^2+^ATPase (SERCA-2A) and β-myosin-heavy chain (β-MHC) genes (Atp2a2 and Myh7, respectively) in murine hearts after one or eight weeks of pressure overload induced by transverse aortic constriction (TAC). As expected, all TAC hearts displayed a significant reduction in SERCA-2A and a significant induction of β-MHC mRNA levels. Interestingly, opposite histone H3 modifications were identified in the promoter regions of these genes after TAC, including H3 dimethylation (me2) at lysine (K) 4 (H3K4me2) and K9 (H3K9me2), H3 trimethylation (me3) at K27 (H3K27me3) and dimethylation (me2) at K36 (H3K36me2). Consistently, a significant reduction of lysine-specific demethylase KDM2A could be found after eight weeks of TAC at the Atp2a2 promoter. Moreover, opposite changes in the recruitment of DNA methylation machinery components (DNA methyltransferases DNMT1 and DNMT3b, and methyl CpG binding protein 2 MeCp2) were found at the Atp2a2 or Myh7 promoters after TAC. Taken together, these results suggest that epigenetic modifications may underlie gene expression reprogramming in the adult murine heart under conditions of pressure overload, and might be involved in the progression of the normal heart towards HF.

## Introduction

Heart failure (HF) represents an end-stage phenotype of a number of cardiovascular diseases, frequently anticipated by cardiac hypertrophy, the earliest and general response of the heart to injury or increased workload [1], [2]. Epidemiological, clinical and experimental data point out that cardiac hypertrophy induced by pathological stimuli is maladaptive and eventually detrimental, and therefore an early therapeutic target to prevent HF [1], [3], [4].

In response to cardiac overload, changes in the gene transcription program are required to obtain de novo synthesis of contractile, structural or regulatory proteins [5], [6], but are also associated to the re-induction of fetal genes including Myh7, the gene encoding for β-myosin-heavy chain (β-MHC), and the repression of post-natal genes such as Atp2a2, the gene encoding for sarcoplasmic reticulum Ca^2+^ ATPase (SERCA-2A) [7], [8]. Such gene expression reprogramming is considered a hallmark of pathological hypertrophy and HF, and its inhibition through different approaches in different animal models has been shown to reduce cardiac hypertrophy and ameliorate cardiac dysfunction [5], [9]. Thus, understanding the molecular mechanisms involved in these processes might represent an important and necessary step to develop new therapeutic approaches for heart disease [10], [11].

The impact of epigenetic mechanisms, i.e. heritable changes in gene expression without alterations in the gene sequence, is now emerging as a major player in cardiovascular pathophysiology [12]. Several epigenetic modifications, including histone modifications can affect the chromosomal structure, DNA accessibility, and in turn gene expression [13], [14]. It has been previously reported that histone acetylation is critical for the induction of hypertrophic changes in cardiac muscle cells by phenylephrine [15]. Consistently, class II histone de-acetylases (HDACs) 5 and 9 exert anti-hypertrophic effects by inhibiting the expression of pro-hypertrophic genes [16].

The presence and extent of DNA or histone methylation have been also recently addressed in murine cardiac hypertrophy [17] or human HF, leading to the identification of specific epigenetic patterns in patients with end-stage HF [18], [19] and the opportunity of defining novel biomarkers of cardiovascular diseases through an epigenetic approach [18], [19]. Although these data are very promising, the map and nature of the epigenetic changes as well as the mechanisms leading to epigenetic alterations in failing hearts are far to be completely elucidated.

In the present study, we hypothesized that epigenetic modifications may underlie transient and/or stable changes of the gene expression program occurring in HF induced by pressure overload. To test this possibility, we used a well-established mouse model of cardiac hypertrophy and HF induced by transverse aortic constriction, and analyzed chromatin marks at the Atp2a2 and Myh7 promoters after one or eight weeks of pressure overload.

## Materials and Methods

### Animal studies

All experiments involving animals were conform to the Guide for the Care and Use of Laboratory Animals published by the US National Institutes of Health (NIH Publication No. 85-23, revised 1996), to the guidelines from Directive 2010/63/EU of the European Parliament and were approved by the animal welfare regulation of University Federico II of Naples, Italy. Wild-type C57/BL6 mice were purchased from the Jackson Laboratory. Male adult mice (age 8 to 9 weeks, N = 50) were included in the study and maintained under identical conditions of temperature (21±1°C), humidity (60±5%) and light/dark cycle, and had free access to normal mouse chow.

### Mouse model of pressure overload

Pressure overload was induced in wild-type C57BL/6 mice (8-week-old; n = 26) by transverse aortic constriction (TAC) as previously described [4]. Mice were anesthetized with an intraperitoneal injection of 0.1 ml/kg of mixture of 50% Tiletamine and 50% Zolazepam (Zoletil 100) and Xylazine 5 mg/kg (Sigma-Aldrich). Another group of animals underwent a left thoracotomy without aortic constriction (SHAM, n = 8). Mice from all the groups were sacrificed one week (1w) or 8 weeks (8w) after surgery to perform molecular analyses. At sacrifice, after body weight (BW) calculation, mice were anesthetized as described above, and the efficacy of the pressure overload was invasively tested by measuring the arterial pressures in the right carotid artery (proximal to the suture) and the left right carotid artery (distal to the suture) or noninvasively by transthoracic Doppler echocardiography. Only TAC animals with systolic pressure gradients >40 mmHg were included in the study. Next, the hearts were removed and cardiac chambers dissected to assess left ventricular weight.

### Transthoracic echocardiography

Cardiac function was non-invasively monitored by transthoracic echocardiography using the Vevo 770 high resolution imaging system (VisualSonics, Toronto, Canada) before the surgery and right before termination, 1w and 8w after surgery. Briefly, the mice were anesthetized with an intraperitoneal injection of 0.1 ml/kg of mixture of 50% Tiletamine and 50% Zolazepam (Zoletil 100) and Xylazine 5 mg/Kg (Sigma-Aldrich), and echocardiograms were performed with a 30-MHz RMV-707B scanning head (Visualsonics, Toronto, Canada).

### RNA extraction and real-time PCR

Total RNA was prepared using TRIzol (Invitrogen, Eugene, OR), according to the manufacturer's instruction. Oligo-dT first strand cDNA were synthesized using the SuperScript VILO cDNA Synthesis Kit (Invitrogen, Life technologies) according to the manufacturer's instructions. mRNA expression was determined in cardiac samples from different experimental groups by real-time quantitative PCR (RT-PCR) using a IQ-5 Multicolor Real-Time PCR Detection System (BIORAD). The primers used were: β-MHC: forward 5′-CGGAAACTGAAAACGGAAAG-3′, reverse 5′-TCCTCGATCTTGTCGAACTTG-3′; SERCA-2A: forward 5′-TCGACCAGTCAATTCTTACAGG-3′, reverse 5′-CAGGGACAGGGTCAGTATGC-3′; GAPDH: forward 5′-TGCAGTGGCAAAGTGGAGATT-3′, reverse 5′- TCGCTCCTGGAAGATGGTGAT-3′. The initial denaturation phase was 5 min at 95°C followed by an amplification phase as detailed below: denaturation at 95°C for 10 s; annealing at 60°C for 30 s; elongation at 72°C for 30 s; detection at 72°C for 40 cycles.

### Quantitative Chromatin Immunoprecipitation (ChIP) assay

Protein bound to DNA was cross-linked by treating tissues of mouse heart with 1% formaldehyde at room temperature, stopping the reaction 10 min later by the addition of 2.5M glycine to a final concentration of 125 mM, followed by 5 min incubation at room temperature. The ChIP assays were performed using the EpiQuik Tissue Chromatin Immunoprecipitation Kit from Epigentek Group Inc. (Brooklyn, NY). Antibodies used for Protein-DNA immunoprecipitation were: anti-FBXL11 (KDM2A; Novus Biologicals, Littleton, CO, USA), anti-dimethyl- H3K36 (H3K36me2; Millipore Corporation, Billerica, MA, USA), anti di-methyl-H3K9 (H3K9me2: Millipore Corporation, Billerica, MA, USA), anti tri-methyl-H3K27 (H3K27me3; Upstate Biotechnology, Dundee, UK), anti-MeCp2 (Abcam Inc., Cambridge, MA, USA), anti-DNMT1 and anti-DNMT3b (Abcam Inc., Cambridge, MA, USA), and normal mouse IgG as a negative control antibody. DNA from these samples was subjected to quantitative PCR analyses, using Power SYBR Green PCR Master Mix (Life Technologies Corporation, Carlsbad, California) in a Chromo4 Real Time thermocycler (BIORAD, London, UK). Amplification of the Atp2a2 promoter fragment was performed using the primers: forward 5′-AGCCAAGGACACCAGTGC-3′ (position from −547 nucleotides to −530) and reverse 5′-GGGATAGAGCGCGGAGTT-3′ (position from −422 nucleotides to −405) amplifying a 143 bp fragment. For amplification of the Myh7 promoter fragment the following primers were used: forward 5′-ACGACCTCCGGATCTGAGT-3′ (position from −75 nucleotides to −57) and reverse 5′-GCGCGCGCTCTTATATAGTT-3′ (position from −34 nucleotides to −15) amplifying a 61 bp fragment. The quantitative PCR conditions were: 95°C for 10 min followed by 40 cycles of 95°C for 15 s, 62°C for 1 min. All PCR signals from immunoprecipitated DNA were normalized to PCR signals from non-immunoprecipitated input DNA. Non-immune Serum or IgG (Mock) was the negative control for the non-specific pull-down of DNA during immunoprecipitation, and provided a measurement of the background or noise for the ChIP system. Results are expressed as percentage (%) of the input with the method described previously [20]. Calculations take into account the values of at least three independent experiments.

### Statistical analysis

Data are expressed as mean ± standard deviation (SD). Comparisons between 2 groups were performed using the unpaired Student t test. Multiple comparisons were made by 2-way analysis of variance (ANOVA) or by 1-way ANOVA with Bonferroni correction and Dunnett's post-test. For all analyses, a minimum value of p<0.05 was considered significant; when present, a p value<0.01 was specified. All the analyses were performed with GraphPad Prism version 5.01 (GraphPad Software, San Diego, CA).

## Results

### Gene expression reprogramming in pressure overload-induced heart failure

To investigate the mechanism(s) involved in left ventricular (LV) gene expression re-programming during cardiac hypertrophy and failure, we analyzed a well-known murine model of cardiac pressure overload induced by transverse aortic constriction (TAC) [4] at two time points: one week (1w) or eight weeks (8w) after surgery, and we focused our study on two representative genes, Atp2a2 and Myh7, whose reprogramming in cardiac hypertrophy and failure is well established [1]. As expected, TAC mice showed a marked increase in LV mass and a significant reduction in LV function, as shown by increased LV weight to body weight (BW) ratio (LVW/BW), and decreased percent fractional shortening (FS%) compared to SHAM (Table S1 in File S1). LV samples from both 1w and 8w TAC mice were characterized by significantly increased expression of β-MHC (Figure S1A in File S1), while SERCA-2A levels were markedly reduced (Figure S1B in File S1).

### Chromatin changes at the Atp2a2 and Myh7 gene promoters in pressure overload-induced heart failure

**Figure 1:**
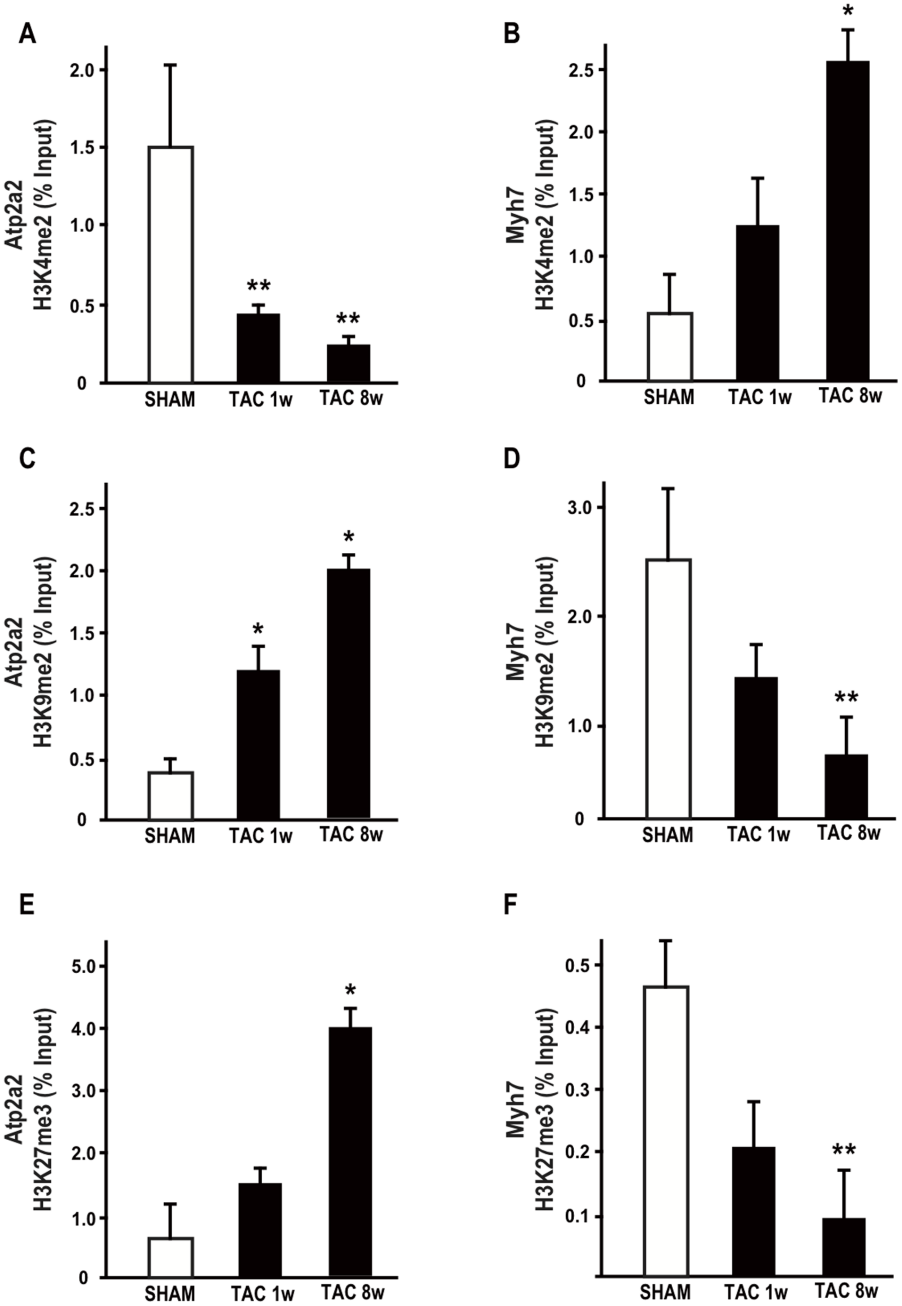
Chromatin modifications at the Atp2a2 and Myh7 gene promoters in murine heart failure. ChIP experiments were performed at the Atp2a2 or Myh7 gene promoters using anti-dimethyl-H3K4 (H3K4me2; **A** and **B**), anti-dimethyl-H3K9 (H3K9me2; **C** and **D**) and anti-trimethyl-H3K27 (H3K27me3; **E** and **F**) antibodies. Each experiment was repeated at least three times, and the quantitative PCR analyses were performed in triplicate. The data are presented as percentages (%) of input DNA (mean ± SD; *p<0.05 vs. SHAM; **p<0.01 vs. SHAM; n = 4–6 hearts/group).

To further investigate whether epigenetic mechanisms may mark gene expression reprogramming occurring in the progression of the normal heart towards HF, we analyzed several different chromatin marks associated with transcriptional activation or repression at the Atp2a2 and Myh7 gene promoters. First, we analyzed the well-established mark of active state, H3 di-methylation (me2) at lysine (K) 4 (H3K4me2) at Atp2a2 and Myh7 gene promoters in SHAM or TAC mice. Chromatin immunoprecipitation analysis (ChIP) demonstrated in both 1w and 8w TAC hearts a remarkable decrease of this mark at the Atp2a2 gene promoter, while a significant increase of H3K4me2 at the Myh7 promoter was observed in 8w TAC (Figure 1, A–B). Conversely, as repressive chromatin marks, we analyzed H3 di-methylation at K9 (H3K9me2) and H3 tri-methylation (me3) at K27 (H3K27me3), and found increased levels of both markers at Atp2a2 promoter. In contrast, at Myh7 promoter, decreased H3K9me2 and H3K27me3 were observed (Figure 1, C–F). Thus, both repressive and activating chromatin modifications at Myh7 and Atp2a2 genes were consistent with the observed gene expression changes (Figure S1 in File S1).

### Recruitment of epigenetic writers, readers and erasers at Atp2a2 and Myh7 gene regulatory regions in TAC hearts

**Figure 2:**
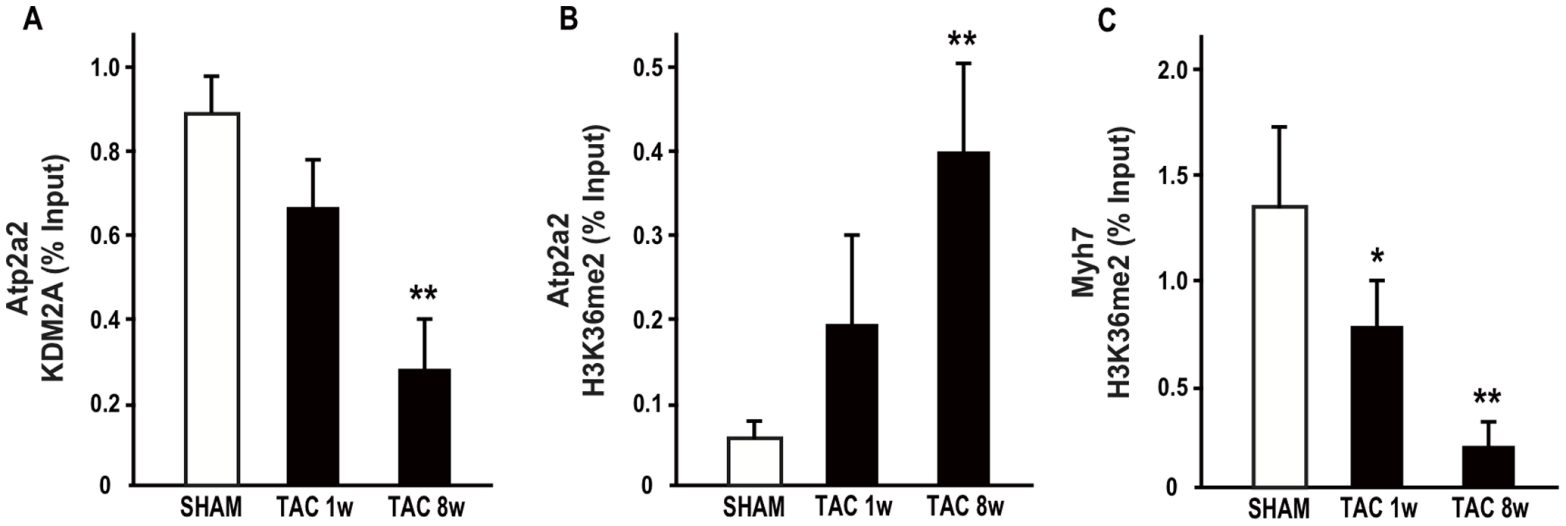
Recruitment of KDM2A and anti-dimethyl-H3K36 (H3K36me2) at Atp2a2 and Myh7 gene promoter regions in TAC hearts. ChIP experiments were performed using antibodies indicated in each panel: **A** KDM2A; **B–C** anti-dimethyl-H3K36 (H3K36me2). Each experiment was repeated at least three times, and the quantitative PCR analyses were performed in triplicate. The data are presented as percentages (%) of input DNA (mean ± SD; *p<0.05 vs. SHAM; **p<0.01 vs. SHAM; n = 4–6 hearts/group).

**Figure 3:**
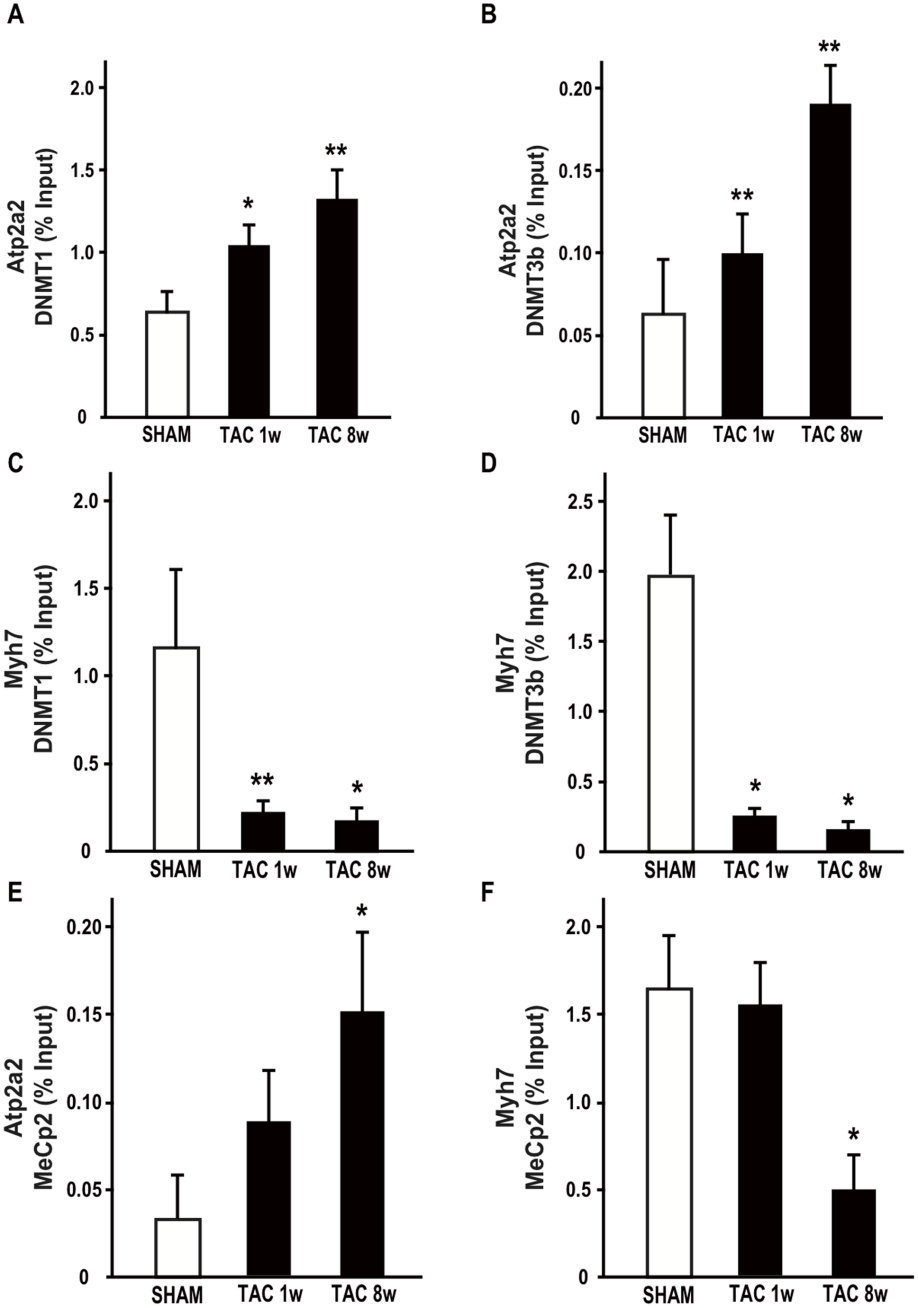
Atp2a2 and Myh7 promoter modifications in pressure overload-induced heart failure. ChIP experiments were performed using antibodies indicated in each panel: **A,C** anti-DNMT1; **B,D** anti-DNMT3b; **E–F** anti-MeCp2. Each experiment was repeated at least three times, and the quantitative PCR analyses were performed in triplicate. The data are presented as percentages (%) of input DNA (mean ± SD; *p<0.05 vs. SHAM; **p<0.01 vs. SHAM; n = 4–6 hearts/group).

**Figure 4:**
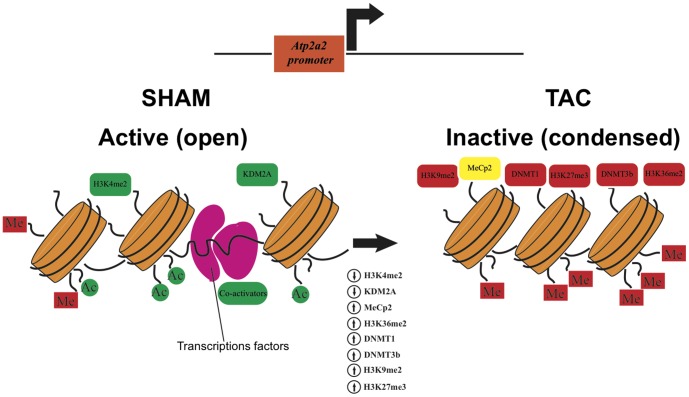
Molecular events underlying the epigenetic switch of the Atp2a2 gene promoter in murine heart failure. Activating chromatin modifiers and modifications are reported in green, while repressive marks are shown in red. DNA methyl-binding proteins are yellow.

The epigenetic dynamics at the Atp2a2 and Myh7 gene promoters were analyzed in further detail, by testing a potential recruitment of epigenetic modifying factors. The first analysis was performed on the histone lysine-specific demethylase 2A (KDM2A), previously shown to be responsible for the maintenance of the unmethylated state of dense CpG islands [21]. In fact, KDM2A may demethylate H3 dimethylation at K36 (H3K36me2), thus preventing the recruitment of DNA methyltransferases to genomic CpG islands. ChIP experiments demonstrated that KDM2A was displaced from Atp2a2 promoter after 8w of pressure overload (Figure 2A). Consistently, H3K36me2 levels were significantly increased in 8w TAC ventricles at the Atp2a2 promoter (Figure 2B), while they were decreased at the Myh7 gene promoter (Figure 2C). ChIP experiments also demonstrated that DNA cytosine-5-methyltransferase 1 (DNMT1) and 3b (DNMT3b) were significantly recruited at the Atp2a2 promoter in both 1w and 8w TAC hearts (Figure 3, A and B), while they were reduced at the Myh7 gene promoter (Figure 3, C and D). In addition, the methyl CpG binding protein 2 (MeCp2), which may be part of the chromatin repressive complex, was recruited 8w after TAC at the Atp2a2 promoter (Figure 3E), while it was significantly reduced in 8w TAC hearts at the Myh7 gene promoter (Figure 3F). Based on these results, a schematic representation of the molecular events underlying the epigenetic transition of the Atp2a2 gene promoter under conditions of pressure overload is presented in Figure 4.

## Discussion

In the present study we demonstrate that transcriptional reprogramming of Atp2a2 and Myh7 genes in pressure overload-induced cardiac hypertrophy and failure is associated with significant epigenetic changes, modifying chromatin dynamics at the promoter regions of these crucial genes. These results suggest that specific chromatin conformational changes might be crucially involved in the progression of the normal heart towards heart failure.

Cardiac hypertrophy requires gene expression reprogramming to promote de novo synthesis of contractile and structural proteins, and usually promotes a “fetal” gene expression program. Since the fetal gene program has been consistently linked to adverse cardiac remodeling, both in humans and in mouse models [22], [23], molecular events that track gene expression re-programming could serve as useful clinical biomarkers of HF progression and as novel potential therapeutic targets.

Several epigenetic modifications, including acetylation, methylation, phosphorylation, ubiquitination, and sumoylation of histone proteins have been shown to modify chromosomal structure and DNA accessibility [13]. Among these, DNA and chromatin modifications orchestrated by DNMTs, chromatin-remodeling complexes and histone-modifying enzymes such as HDACs and histone acetyl transferases (HATs) have been shown to regulate cardiac gene expression during development and in response to pathological stimuli [12], [17], [24].

In recent years, histone acetylation has been largely investigated in different experimental models of cardiac hypertrophy and failure [12], [25]. In contrast, the role of histone and DNA methylation in the heart remains much less defined. While histone acetylation is in general associated to an active state of the chromatin, the effects of histone methylation may be associated with either transcriptional activation or repression, depending on the lysine modified and whether this residue is mono-, di- or tri-methylated [26], [27]. Indeed, while H3 di- and tri-methylation on K9 and K27 (H3K9me2 and H3K27me3) are well established epigenetic marks associated with closed chromatin, di-methylation of H3 on K4 (H3K4me2) is associated with an active chromatin state.

Interestingly, distinct genome-wide patterns of H3K36me3 enrichment have been identified in end-stage human HF or normal hearts [18], [28], suggesting that chromatin methylation may represent a crucial signal in the regulation of gene expression in the heart. Previous work has specifically suggested an involvement of histone methylation in cardiac hypertrophy [29] and, more recently, a global genome study has clearly shown that a specific epigenetic signature, defined by histone acetylation and methylation, regulates the expression of a large set of genes in murine cardiac hypertrophy [17].

Here we show that expression reprogramming of the Atp2a2 and Myh7 genes in adult murine failing hearts is associated with remarkable, opposite epigenetic modifications at their promoter regions. Significant changes of specific chromatin marks, including H3K4me2, H3K9me2, and H3K27me3 were identified at both genes promoter regions. All the observed chromatin changes were consistent with Atp2a2 gene repression and Myh7 reactivation. In particular, H3K27me3 may also favor the recruitment of DNA methyltranferases and may associate with more stable changes in the expression program [32]. Indeed, it has been shown that the susceptibility to DNA methylation of CpG islands is strictly dependent on K36me2 levels and recruitment of KDM2A. KDM2A promotes K36me2 demethylation, thus specifically erases this chromatin mark and allows CpG rich promoters to remain non-methylated [33]. Nucleation of KDM2A at CpG dense regions results in removal of H3K36 methylation, creating CpG island chromatin that is uniquely depleted of this modification [33]. Most chromatin changes at Atp2a2 and Myh7 promoter genes identified eight weeks after TAC could also be found as early as one week, even if early modifications have lower magnitude and not always reach statistical significance.

Atp2a2 promoter is embedded in a very dense CpG island, and silencing of SERCA-2A in murine HF is associated with significant loss of KDM2A and increase of K36me2 levels. We hypothesize that this chromatin switch at a CpG rich promoter, together with the increase of K27me3 levels and the recruitment of DNA methylation machinery components (DNMT1, DNMT3a and MeCp2) may represent a “high risk” epigenetic state predisposing to detrimental DNA hypermethylation of Atp2a2 gene and consequent stable repression. In fact, hypermethylation of several genes has been observed in end-stage human HF [28].

While other groups performed their analysis in cardiomyocytes isolated from TAC hearts [17], in our study we investigated the extent and role of epigenetic changes in whole TAC hearts. Importantly, our approach suffers some limitations, mainly due to the impossibility to distinguish between cardiomyocytes and other cardiac cell types, especially fibroblasts that might be significantly activated and represented in long-term pressure overload-induced heart failure. However, the isolation and analysis of cardiomyocytes from TAC hearts, while ideally representing the best procedure to analyze epigenetic changes in this cell type, might also produce epigenetic modifications *per se*, and change the epigenetic status in comparison to the living heart. Indeed, it is now widely recognized the epigenetic reprogramming could be even initiated and perturbed by luminous stimuli [30] or permanence in culture can itself modify epigenetic conditions [31].

In conclusion, this study demonstrates that specific epigenetic modifications underlie gene expression reprogramming of Atp2a2 and Myh7 gene promoters in the adult murine left ventricles under conditions of chronic pressure overload, and might be involved in the progression of the normal heart towards HF. Further investigation will be necessary to establish whether the observed chromatin switch in HF, loss of KDM2A and enrichment of H3K36me2 at CpG islands may represent a general mechanism operating in the transition of gene promoters from an unmethylated to a methylated state. In this case, the proposed mechanism would be critical to identify novel potential targets and new emerging epigenetic drugs in the therapy of HF.

## Supporting Information

File S1 **Table S1 & Figure S1.** Table S1. Morphometric and echocardiographic evaluation of mouse hearts from different experimental groups. Figure S1. β-MHC and SERCA-2A mRNA levels in failing murine hearts β-MHC (A) and SERCA-2A (B) mRNA levels in murine hearts after 1 week or 8 weeks of transverse aortic constriction (TAC 1w and TAC 8w, respectively)(*p<0.05 vs. SHAM).
(PDF)
