# Supplementary material for: Epigenetic Switch at Atp2a2 and Myh7 Gene Promoters in Pressure Overload-Induced Heart Failure
Source: PLoS One. 2014 Sep 2;9(9):e106024. doi: 10.1371/journal.pone.0106024 (PMC4152141; doi:10.1371/journal.pone.0106024)
Supplement: File S1 — Table S1 & Figure S1. Table S1. Morphometric and echocardiographic evaluation of mouse hearts from different experimental groups. Figure S1. β-MHC and SERCA-2A mRNA levels in failing murine hearts β-MHC (A) and SERCA-2A (B) mRNA levels in murine hearts after 1 week or 8 weeks of transverse aortic constriction (TAC 1w and TAC 8w, respectively)(*p<0.05 vs. SHAM). (PDF) [file pone.0106024.s001.pdf]

Table S1. Morphometric and echocardiographic evaluation of mouse hearts from different experimental groups

	SHAM (n=8)	TAC 1w (n=6)	TAC 8w (n=20)
**Heart morphometry**			
BW (g)	21.0 ± 0.2	23.5 ± 1.6	21.6 ± 0.5
LVW/BW (mg/g)	3.6 ± 0.1	4.8 ± 0.7*	6.2 ± 0.1*
**Echocardiography**			
LVEDd (mm)	3.2 ± 0.3	-	3.6 ± 0.4*
LVESd (mm)	1.4 ± 0.2	-	2.2 ±0.3*
FS (%)	54.7 ± 1.3	-	36.1 ± 2.4*
HR (bpm)	525 ± 20	-	518 ± 15

Abbreviations used: SHAM, sham operated mice; TAC 1w, 1 week of transverse aortic constriction; TAC 8w, 8 weeks of transverse aortic constriction; BW, body weight; LVW, left ventricle weight; LVEDd, left ventricle end-diastolic diameter; LVESd, left ventricle end-systolic diameter; FS, fractional shortening; HR, heart rate; *p<0.001 vs. SHAM.

Figure S1

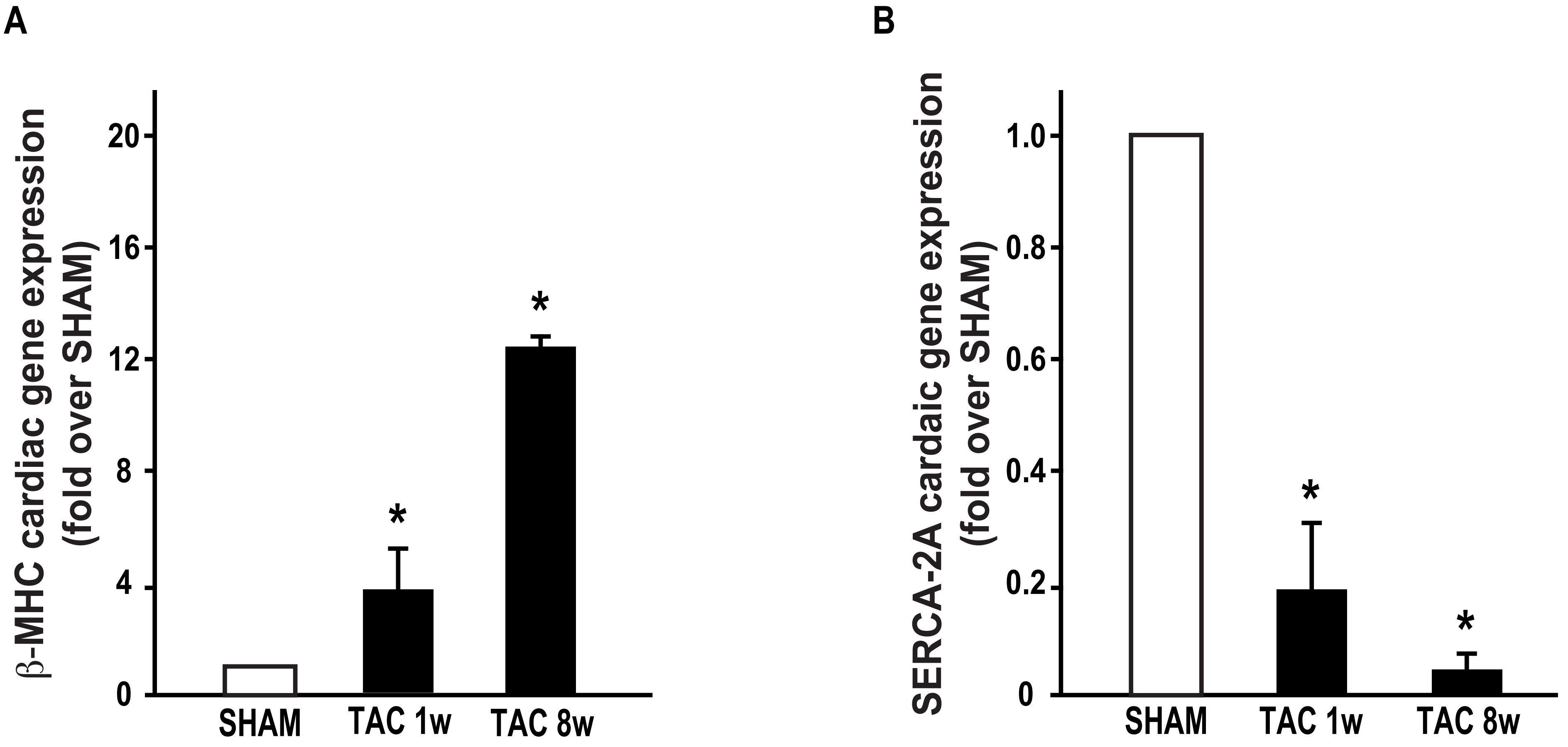

Figure S1. **β-MHC and SERCA-2A mRNA levels in failing murine hearts**
β-MHC (**A**) and SERCA-2A (**B**) mRNA levels in murine hearts after 1 week or 8 weeks of transverse aortic constriction (TAC 1w and TAC 8w, respectively)(*$p<0.05$ vs. SHAM).
